# Brain Neuronal CB2 Cannabinoid Receptors in Drug Abuse and Depression: From Mice to Human Subjects

**DOI:** 10.1371/journal.pone.0001640

**Published:** 2008-02-20

**Authors:** Emmanuel S. Onaivi, Hiroki Ishiguro, Jian-Ping Gong, Sejal Patel, Paul A. Meozzi, Lester Myers, Alex Perchuk, Zoila Mora, Patricia A. Tagliaferro, Eileen Gardner, Alicia Brusco, B. Emmanuel Akinshola, Bruce Hope, Javier Lujilde, Toshiya Inada, Shinya Iwasaki, David Macharia, Lindsey Teasenfitz, Tadao Arinami, George R. Uhl

**Affiliations:** 1 Department of Biology, William Paterson University, Wayne, New Jersey, United States of America; 2 Molecular Neurobiology Branch, Intramural Research Program, National Institute on Drug Abuse (NID)-National Institutes of Health (NIH), Bethesda, Maryland, United States of America; 3 Department of Medical Genetics, Institute of Basic Medical Sciences, University of Tsukuba, Japan; 4 Facultad de Medicina, Universidad de Buenos Aires, Buenos Aires, Argentina; 5 Department of Pharmacology, Howard University, Washington, D. C., United States of America; 6 Behavioral Neuroscience Branch, Intramural Research Program, National Institute on Drug Abuse (NID)-National Institutes of Health (NIH), Bethesda, Maryland, United States of America; 7 Chiba Medical Center, Teikyo University, Ichihara, Chiba, Japan; University of Sydney, Australia

## Abstract

**Background:**

Addiction and major depression are mental health problems associated with stressful events in life with high relapse and reoccurrence even after treatment. Many laboratories were not able to detect the presence of cannabinoid CB2 receptors (CB2-Rs) in healthy brains, but there has been demonstration of CB2-R expression in rat microglial cells and other brain associated cells during inflammation. Therefore, neuronal expression of CB2-Rs had been ambiguous and controversial and its role in depression and substance abuse is unknown.

**Methodology/Principal Findings:**

In this study we tested the hypothesis that genetic variants of *CB2* gene might be associated with depression in a human population and that alteration in *CB2* gene expression may be involved in the effects of abused substances including opiates, cocaine and ethanol in rodents. Here we demonstrate that a high incidence of (Q63R) but not (H316Y) polymorphism in the *CB2* gene was found in Japanese depressed subjects. CB2-Rs and their gene transcripts are expressed in the brains of naïve mice and are modulated following exposure to stressors and administration of abused drugs. Mice that developed alcohol preference had reduced *CB2* gene expression and chronic treatment with JWH015 a putative CB2-R agonist, enhanced alcohol consumption in stressed but not in control mice. The direct intracerebroventricular microinjection of CB2 anti-sense oligonucleotide into the mouse brain reduced mouse aversions in the plus-maze test, indicating the functional presence of CB2-Rs in the brain that modifies behavior. We report for the using electron microscopy the sub cellular localization of CB2-Rs that are mainly on post-synaptic elements in rodent brain.

**Conclusions/Significance:**

Our data demonstrate the functional expression of CB2-Rs in brain that may provide novel targets for the effects of cannabinoids in depression and substance abuse disorders beyond neuro-immunocannabinoid activity.

## Introduction

Drug addiction and major depression are mental health problems associated with stressful events in life with high relapse and reoccurrence even after treatment [1]. Major depression is characterized by mood changes and anhedonia. Anhedonia is a lack of interest in pleasurable things of life and can be studied using the chronic mild stress (CMS) model of depression in rodents [2]. Like depression, it is recognized that drug addiction is a brain disease [3]. Significant effort has been made to uncover genetic markers for substance abuse and depression [4], [5]. One rationale for use of abused substances including marijuana is the self-medication hypothesis. Evidence for an association between cannabis use and depression has grown [1]. Comorbid presentation of cannabis abuse and depression is common [4]. Studies suggest that cannabis abuse in adults increases depressive symptoms, but depression does not predict later cannabis abuse [6], [7]. The discovery of an endocannabinoid physiological control system (EPCS) [8], has led to the examination of this system in CNS and its role in mental disorders [4]. Thus a role of the EPCS in a number of neuropsychiatric disorders has been described [5]. Two receptors are activated by cannabinoids or marijuana use [8]. CB1-Rs are expressed in brain and periphery, while CB2-Rs were thought to be expressed in immune cells and were referred to as peripheral CB2-Rs. However, the neuronal expression of CB2-Rs in the brain and its role in depression and substance abuse is unknown. While a number of laboratories were not able to detect the presence of CB2-Rs in healthy brains [9]–[11], there has been demonstration of CB2-R expression in rat microglial cells and other brain associated cells during inflammation [12]–[17]. Preliminary report of some of the data have been presented as abstracts at scientific conferences and described in summary form in a recent general review paper [18]. We have also reported the involvement of cannabinoid CB2-Rs in alcohol preference in mice and alcoholism in humans [19], which supports the functional presence of neuronal CB2-Rs in the mammalian CNS.

With novel and precise cannabinoid probes, our results indicate the expression of brain CB2-Rs in mouse model of depression and in the effects of abused substances [20]. We and others have now identified and reported the presence of CB2-Rs in brain neuronal and glial process [20]–[24]. To further improve understanding of the role of CB2-Rs in the brain, we hypothesized that genetic variants of *CB2* gene might be associated with depression in a human population and that alteration in *CB2* gene expression may be involved in the effects of abused substances in rodents. Our data reveals that CB2-Rs are expressed in brain and plays a role in depression and substance abuse.

## Results

### Involvement of *CB2* gene expression in depression and in the effects of abused drugs

**Figure 1:**
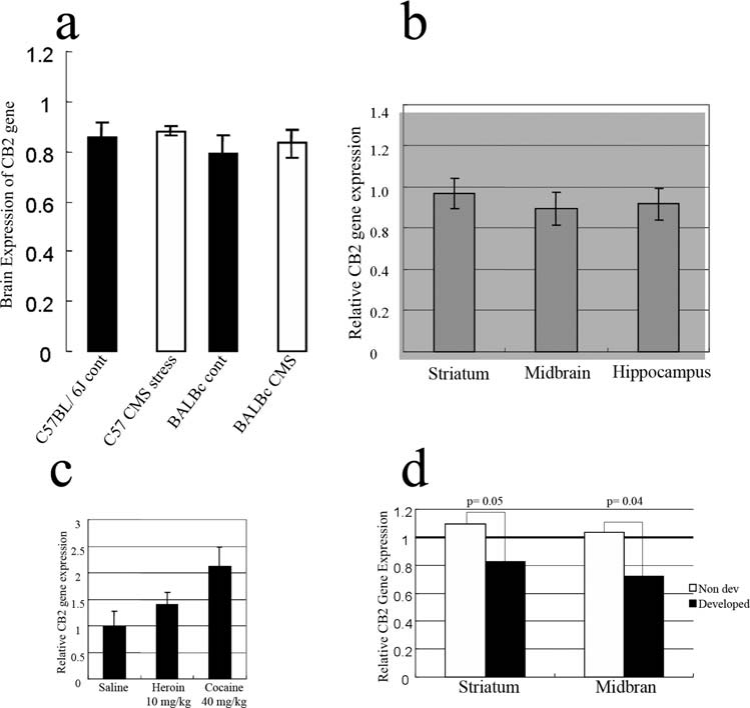
Presence of *CB2* gene in the brain. A, Relative brain expression of *CB2* gene in C57BL/6J and BALBc strains subjected to stress. *B*, Relative *CB2* gene expression levels in the striatum, midbrain, and hippocampus of C57Bl/6J mice. *C*, Mouse whole brain relative *CB2* gene expression levels following chronic treatment with heroin and cocaine. *D*, relative *CB2* gene expression levels in striatum and midbrain of mice that developed alcohol preference. *CB2* gene expression was relative to the standard laboratory brain obtained from C57BL/6J that was set to 1.0. The positive control was from the spleen and no cDNA in TaqMan PCR reaction served as negative controls.

We first determined *CB2* gene expression in mice brains exposed to chronic mild stressors or those treated chronically with abused substances like heroin, cocaine, or those on the alcohol consumption preference test [22]. *CB2* gene was expressed in whole brain preparations of CMS and control mice (Fig. 1A). *CB2* gene expression was then confirmed in different mouse brain areas including striatum, midbrain and hippocampus (Fig. 1B). In both figures 1 A and B, the control samples were set at 1.0 and the spleen was used as positive controls because it has the most abundant expression of CB2-Rs in mammals. These results showed that *CB2* gene is present and expressed in the brains of naïve mice and in those exposed to chronic mild stress.

**Figure 2:**
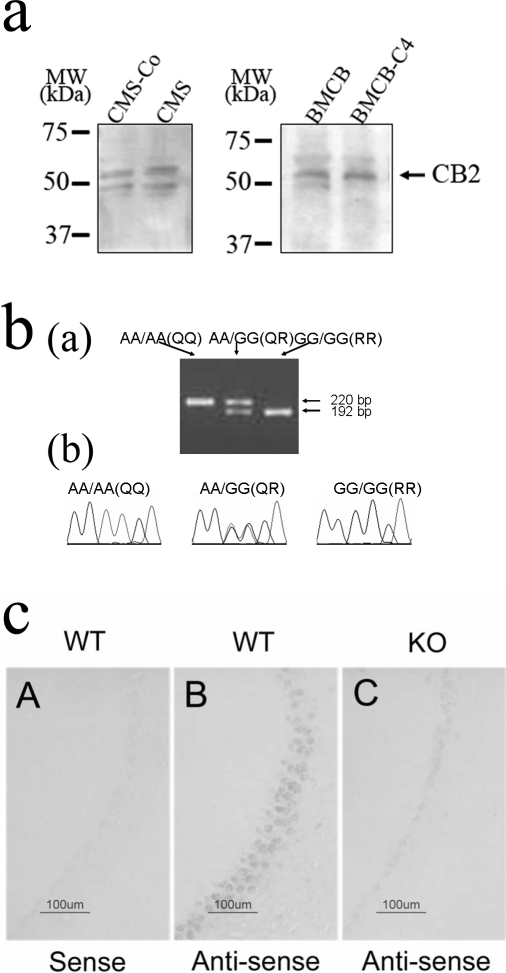
Brain CB2-Rs: Immunoblots, genotyping and *in-situ* hybridization. *A*, *In-situ* hybridization indicating *CB2* gene is expressed in the cerebellum of wild type and not in the cerebellum of the CB2-R deficient mice and also in sense controls in the wild type mice. *B*, RFLP genotyping discrimination on agarose gel for *CB2* Q63R polymorphism in depressed subjects (Ba) and, Resequences of *CB2* Q63R polymorphism (Bb). *C*, Western blotting of CB2-Rs in CMS and control mice (left panel) and in right panel in mice exposed to 4 mg/kg capsaicin in utero.

We then determined more precisely the involvement of *CB2* gene expression in separate groups of mice chronically treated with heroin (10 mg/kg) or cocaine (40 mg/kg) or those exposed to varying alcohol consumption. Chronic treatment with heroin increased (p>0.05) while cocaine significantly (p<0.05) increased *CB2* gene expression in mouse brain preparations using RT-PCR (Fig. 1C). In mice subjected to the chronic varying alcohol intake paradigm for alcohol preference, there was significant reduction *CB2* gene expression in the striatum (P = 0.05) and ventral midbrain (p = 0.04), whereas in mice with little preference for drinking alcohol, there were no changes in *CB2* gene expression in these brain regions (Fig. 1D). The alcohol data support our previous studies [19] that CB2-R agonist JWH015 administration enhances alcohol intake in stressed but not in non-stressed control mice. In contrast the administration of the CB2-R antagonist AM630 reduced alcohol intake (P = 0.08) in stressed but had no effect in the alcohol consumption in non-stressed naive mice. The presence of CB2-Rs in the brain was further investigated in CB2-R deficient mice and their wild type litter mates. *In-situ* hybridization data show that *CB2* gene is expressed in the cerebellum of wild type and not in the cerebellum of the CB2-R deficient mice and also in sense controls in the wild type mice (Fig. 2C). Altogether, these results revealed the functional presence of brain CB2-Rs that plays a role in the effects of abused substances.

We then examined the association between *CB2* gene polymorphism and depression in a human population to test the hypothesis that genetic variants of *CB2* gene might be associated with depression and substance abuse in Japanese population. A significant association was found between the *CB2* Q63R polymorphism and Japanese depressed subjects (p = 0.007, odds ratio 1.42, 95% confidence interval: 1.09–1.831), (Fig. 2B). Furthermore, because a previous study showed a significant functional difference of RR genotype in lymphocyte, we compared the distribution of subjects without or with Q allele. The RR genotype was significantly associated with depression [p = 0.01, odds ratio 1.95; 95% confidence interval, (1.11–3.4402.28)], (Table 1).

**Table 1:** Allelic and genotype distribution of R63Q polymorphism in the *CB2 gene*.

		genotype				allele	
major depression	RR	RQ	QQ	total	GG	AA	total
n	65	85	16	166	215	117	332
%	0.39	0.51	0.10		0.648	0.325	
controls	RR	RQ	QQ	total	GG	AA	total
n	147	256	84	487	550	424	974
%	0.30	0.53	0.17		0.565	0.435	
	RR vs RQ+QQ: p = 0.01				allelic comparison: *p* = 0.0067 OR = 1.42 (1.09–1.83		
					(Two-sided)		

Comparisons were made between patients with major depression and the healthy controls. Significant differences are observed in allelic frequency and genotype distribution in the R recessive model. OR is odd ratio.

### Analysis of CB2-Rs in the rodent brain with or without exposure to stressors

**Figure 3:**
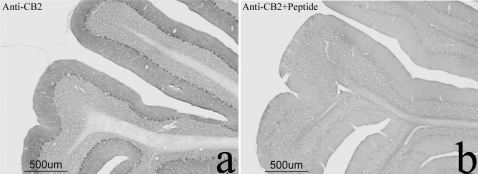
Brain CB2-Rs: Immunoractivity (IR) and pre-adsorption with immunizing peptide. *A*, CB2-IR in the left panel and lack of CB2-IR in the right panel, *B*, when the CB2 antibody was pre-adsorped with the peptide.

To determine the localization of CB2-Rs in mouse and rat brains, we used a combination of Western blotting, immunohistochemistry and *in-situ* hybridization. The CB2-R knockout mice and their wild type litter mates were included as controls for the *in-situ* hybridization. We then analyzed CB2-Rs in the brains of mice subjected to chronic mild stressors, including adult mice that had been prenatally exposed to capsaicin. Western blotting analyses from mice brains revealed a major CB2-R band of approximately 53 kDa (Fig. 2A), with other visible bands around 37 kDa and 75 kDa, similar to those reported [21]. *CB2 gene* was expressed in mouse whole brain preparations and the CB2-R protein was also present in the CMS and prenatal capsaicin exposure (Fig. 2A). The specificity of three commercial CB2-R antibodies had been examined in our previous studies to map CB2-R immunoreactivity in the rat brain [24]. In this study the specificity of the CB2-R antibody used was further confirmed as the CB2-R immunoreactivity detected in the cerebellum were undetectable when the CB2-R antibody was pre-adsorbed with the immunizing peptide (Fig. 3A and B) using 8.3 µg/ml of the CB2 sequence peptide used to produce the antiserum. It is important to note that we previously demonstrated and reported that CB2-R immunoreactivity was present in the CA2 region of the hippocampus, spleen and interpolar part of spinal 5^th^ nucleus of wild type brain and the CB2-R immunoreactivity was absent in these structures in the global CB2-R knockout mouse [24].

**Figure 4:**
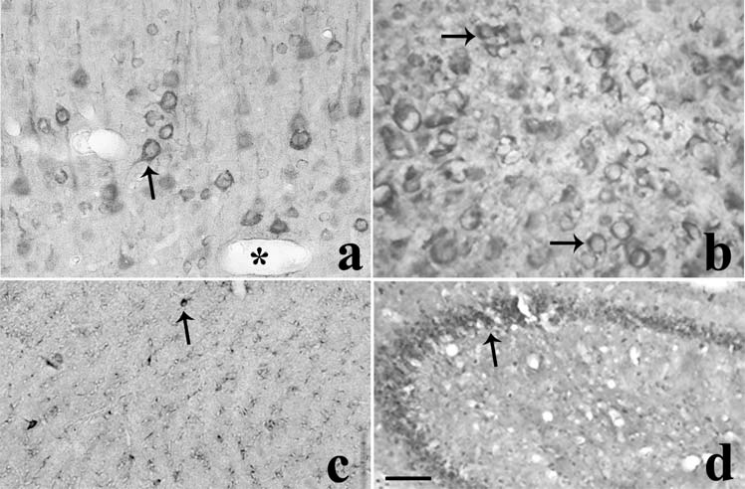
Brain CB2-Rs: Immunohistochemistry in mouse and rat brain. *A*, CB2-IR in apical dendrites and cell bodies of pyramidal neurons of rat cerebral cortex. *B*, CB2-IR in mouse cerebral cortex. *C*, CB2-IR in rat corpus callosum and *D*, CB2-IR in mouse hippocampal allocortex and some interneurons in the striatum oriens and stratum radiatum.

We then performed immunohistochemical analysis in the naïve mouse and rat brain sections (Fig. 4). Apical dendrites and cell bodies of pyramidal neurons of rat cerebral cortex were moderately to heavily immunolabeled for CB2-R. Scattered fibers in the rat cerebral cortex showed CB2-R-IR (Fig. 4A). CB2-R immunoreactivity (IR) was also observed in the mouse cerebral cortex (Fig. 4B). CB2-R-IR was also observed in rat corpus callosum (Fig. 4C). A moderate to dense CB2 immunostaing was observed in pyramidal neuron of mouse hippocampal allocortex and some interneurons in the stratum oriens and stratum radiatum (Fig. 4D). Some glial cells were also immunolabeled for CB2-Rs in the hippocampus (Fig. 4D). This localization pattern is in agreement with the perfect overlay when double labeling of CB2-Rs and neuron specific enolase (NSE) in hippocampal neuronal cultures was visualized by confocal immunofluorescence imaging [24]. Thus, in the brain areas analyzed CB2-R immunoreactivity was detected in mice and rat brains, and this is supported by reports of identification of neuronal CB2-Rs in the brain stem involved in emesis [21].

### Behavioral effects of CB2-R activation and blockade

**Figure 5:**
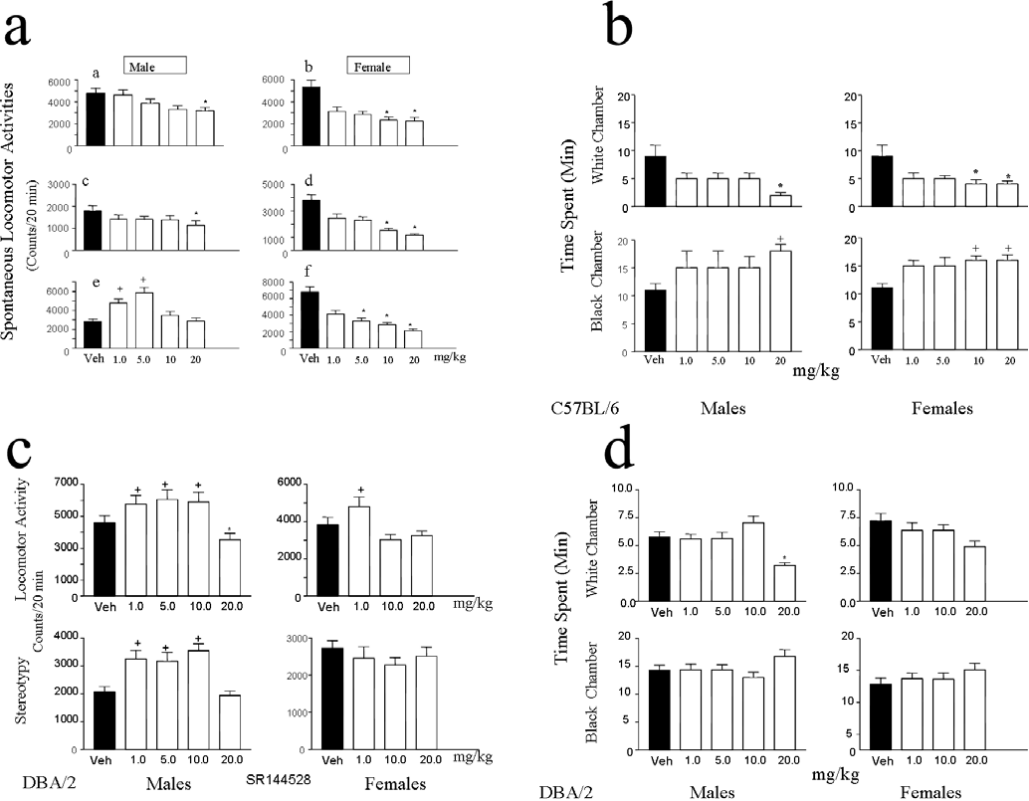
Behavioral effects of CB2-R activation and blockade. *A*, Mouse spontaneous locomotor activity following acute treatment with CB2 agonist JWH015 (1–20 mg/kg), in mouse strain, C57Bl/6 (a and b); BALBc, (c and d) and DBA/2 (e and f). *B*, Effect of JWH015 in C57Bl/6 mice in the two compartment black and white box, showing time spent in the black and white chamber. *C*, Acute effects of SR144528 – a CB2-R antagonist on DBA/2 mouse spontaneous locomotor activity and stereotype behavior. *D*, Acute effects of SR144528, in DBA/2 male and female mice in the two chamber black and white test box, showing time spent in the black and white chamber.

If there are functional CB2-Rs in neurons in the brain as new reports demonstrates [18]–[24], then activation and blockade of CB2-Rs may influence behavior. We therefore examined the behavioral effects of acute activation and blockade of CB2-Rs using measures of locomotor activity, time spent in the two- compartment black and white box and in food consumption tests in mice. JWH015 (1–20 mg/kg) a CB2-R agonist, altered mouse locomotor activities in a strain and gender dependent fashion in three mouse strains (Fig. 5A, a–f). Increasing doses of JWH015 in this study reduced the activity of the animals in general, similar to the report [25] with another CB2-R agonist, GW405833. We also previously reported a similar profile of decreased motor function as demonstrated by the reduction in stereotypy following the administration of the JWH015 compound the three mouse strains [18]. This is in support of the strain and gender depression of motor function with the female sensitive than the male mice. The next sets of experiments were then performed in selected mouse strains. In the two- compartment black and white test box, acute treatment with JWH015 (1–20 mg/kg) induced an anxiogenic profile of response (Fig. 5B), with the females of the C57BL/6 strain more sensitive to the aversive behavior in the white chamber. This response was characterized by a decrease in time spent in the white chamber and a concomitant increase in time spent in the black chamber (p<0.05). Acute administration of SR144528 (1–20 mg/kg), a CB2-R antagonist enhanced (P<0.05) the locomotor activity and stereotype behavior in the DBA/2 strain in comparison to vehicle treated controls. The males were more susceptible to locomotor activation by the acute treatment with CB2-R antagonist than the female mice (Fig. 5C). In contrast to the effects of the agonist JWH015, acute treatment with the antagonist SR144528 (1–20 mg/kg) had little or no effect on the time the DBA/2 strain spent in both chambers of the two- compartment black and white box by both the male and female mice except a reduced time (p<0.05) spent in the white compartment by the male mice at the highest dose (20 mg/kg) used in this study (Fig. 5D). The influence of CB2-R ligands on food consumption was also investigated. The enhancement and suppressant effects of CB2-R ligands were strain and time dependent (data are not shown). Thus, there was a clear strain and gender dependent effects following CB2-R activation or inhibition on behavioral responses as measured by locomotor activity, emotionality and food consumption tests.

### CB2-R gene targeting by CB2 antisense oligonucleotide modifies behavior

**Figure 6:**
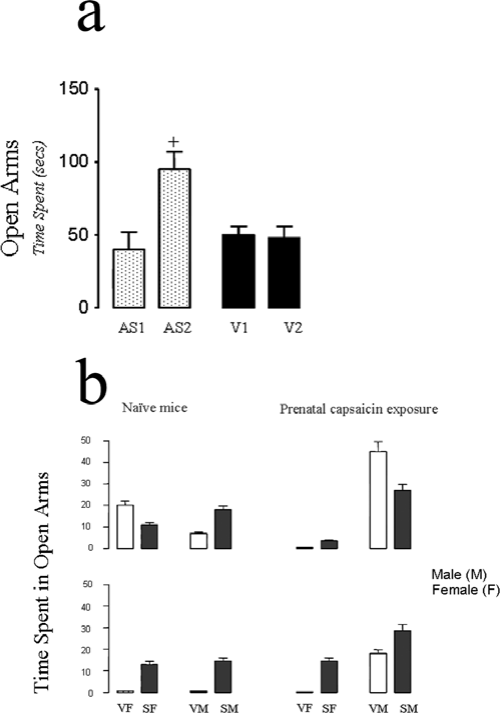
CB2-R gene targeting modifies behavior. *A*, Behavioral effects of CB2 intracerebral gene targeting by antisense oligonucleotide microinjected into the mouse brain and performance of mice in plus-maze test was assessed before and after 3 days of twice daily microinjection. AS1 and AS2 were before and after CB2 antisense oligo microinjection. V1 and V2 are controls. *B*, performance in plus-maze test following CMS or mice exposed prenatally to capsaicin and the effect of JWH015 (20 mg/kg).

We have previously characterized the effects of peripherally administered cannabinoids in the plus maze test of anxiety using mice and rats [26]. To investigate whether *CB2-R* gene targeting by CB2-R antisense oligonucleotide (oligo) modifies behavior, we determined whether inhibition of *CB2* gene expression in the brain will alter mouse behavior in the elevated plus-maze test. The direct intracerebroventricular (ICV) CB2-R antisense oligo (20 µg in 5 µl) microinjection bilaterally into the mouse lateral ventricles significantly reduced mouse aversions (P<0.05) to the open arms of the plus maze (Fig. 6A). In contrast the performance of mice microinjected with the sense and mismatched oligos were not different from the control mice. Other groups of mice that had been exposed to stress by chronic mild stressors for 4 weeks or by prior prenatal exposure to capsaicin were also tested on the plus-maze after acute treatment with intraperitoneal (ip) injection of JWH015 (20 mg/kg). Stress whether by CMS or prenatal exposure to capsaicin induced gender specific aversions in the plus-maze test which was significantly reduced (p<0.05) by JWH015 (Fig. 6B). These data together with the cerebral microinjection of CB2-R antisense oligo that reduced mouse aversions to the open arms of the plus-maze provides further evidence for the functional presence of CB2-Rs in the brain that influence behavior.

### Effects of CB2-R activation and blockade on anhedonia induced by chronic mild stress (CMS)

**Figure 7:**
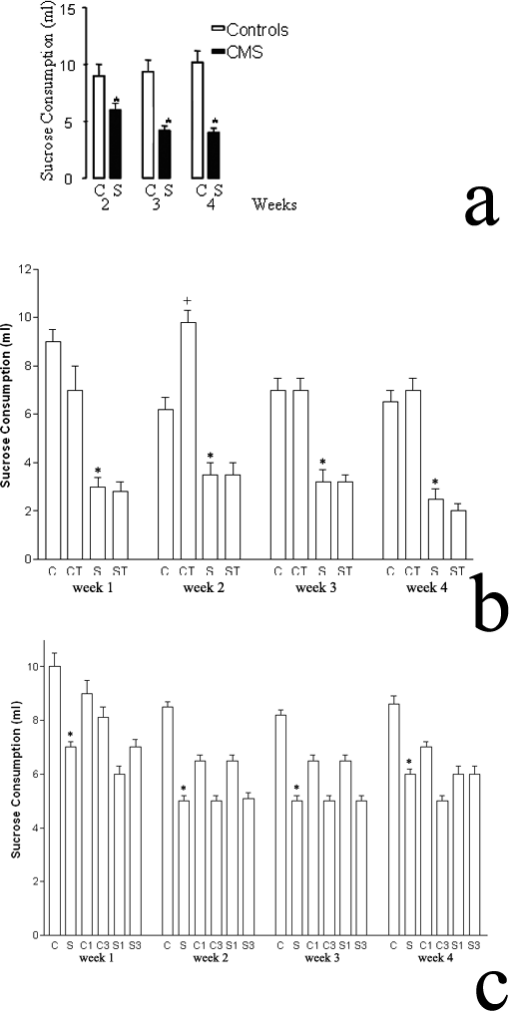
CB2-R- activation and blockade on anhedonia induced by chronic mild stress (CMS). *A*, Weekly sucrose consumption in stress and control mice. *B*, Effect of JWH015 (20 mg/kg) on mouse weekly sucrose consumption test. C, Effect of AM630 (1 and 3 mg/kg) on mouse weekly sucrose consumption test.

In separate experiments we investigated the effects of selected CB2-R agonist or antagonists on anhedonia induced by chronic mild stress. There was no difference in the amount of water that both the CMS and control animals drank in the weekly over night water consumption test, indicating that stress did not interfere with water intake of the animals. In contrast after anhedonia was established by the CMS regime, there was significant reduction (P<0.05-P<0.01) in the amount of sucrose solution consumed by the CMS mice in comparison to control animals in the weekly over night sucrose consumption test (Fig. 7A). The establishment of anhedonia (lack of pleasure) is one of the major validated endpoints in the model of depression using rodents [2]. We then investigated the effects of daily treatment with selected doses of the CB2-R agonist JWH015 (20 mg/kg) or the CB2-R antagonist AM630 (1 and 3 mg/kg) in the CMS and control mice. JWH015 induced variable consumption of sucrose solution in CMS and control mice (Fig. 7B). Stressed mice chronically treated with JWH015 did not differ in their consumption of sucrose solution from the CMS animals that were not treated. Curiously however consumption of sucrose solution was enhanced in control mice (p<0.05) treated daily with JWH015 by week 2 and 4. In contrast to the effects of the CB2-R agonist JWH015, the antagonist AM630 did not significantly modify the intake of sucrose solution in the CMS or in the control animals (Fig. 7C). It is to be noted however, that we have previously shown that alcohol intake was dramatically enhanced in stressed mice treated with JWH015 and that the stressed enhanced alcohol consumption was blocked by the CB2-R antagonist AM630 (p>0.05) [19]. Thus on the basis of alcohol consumption in CMS mice, and the augmentation of alcohol consumption by treatment with the CB2-R agonist and blockade of the stressed induce alcohol consumption by treatment with the CB2-R antagonist, along with the Q63R polymorphism in human alcoholics and depressed subjects, we suggest that CB2-Rs plays a role in substance abuse and depression.

### Subcellular localization of CB2-Rs in the rodent brain

**Figure 8:**
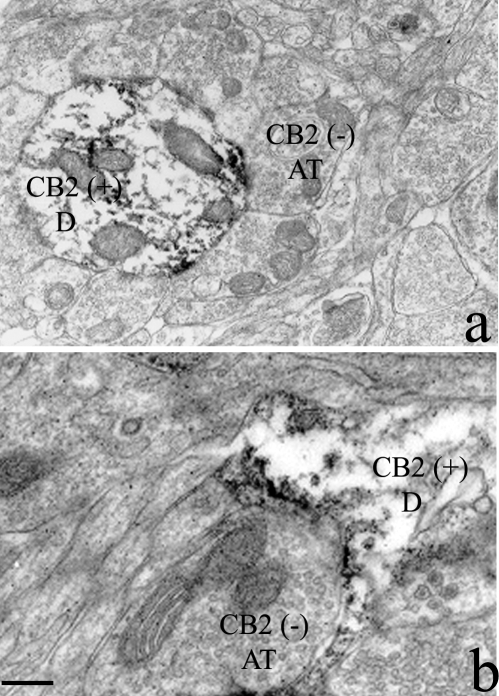
Subcellular localization of rat hippocampal CB2-Rs. *A*, a CB2-IR dendrite [CB2(+) D] receiving multiple synaptic contacts from axon terminals lacking CB2-R immunolabeling [CB2(−) AT]. B, a CB2-IR dendrite [CB2(+) D] was contacted by a non-immunoreactive axon terminal [CB2(−) AT]. Scale bar represents 0.3 µm.

We performed an immunoelectron microscopy study to determine the subcellular localization of CB2-R in the selected rodent brain structures that we have shown contain CB2-R immunoreactivity [23]. CB2-R-IR was observed mostly in dendrites near the plasma membrane and close to the area of contact with axon terminals (Fig. 8). Some CB2-R immunoreactive dendrites were seen to receive multiple synaptic contacts from axon terminals lacking CB2-R-IR (Fig. 8A). In some areas, a CB2 immunoreactive dendrite was contacted by a non-immunoreactive axon terminal (Fig. 8B). This pattern of immunostaining on dendrites and cell bodies indicates a post-synaptic localization in the areas that were analyzed (hippocampus and cerebral cortex). Therefore our results further confirm the presence of CB2-Rs in neuronal structures in the central nervous system.

## Discussion

There is little or no information about the role of CB2-Rs in depression and addictive disorders. Indeed our studies provide the first evidence for a role of CB-Rs in depression and substance abuse. These findings are of importance as it opens new areas of research and approaches in understanding depression and addictive disorders for which pharmacological treatment has been disappointing. Reports by our group [18]–[20], [23], and others [21]–[23], have identified the functional presence of CB2-Rs in neuronal and glial processes contrary to the view that the CB2-Rs were restricted to peripheral tissues and predominantly in immune cells. We also found differential modification of *CB2* gene expression in brain regions of animals treated with abused substances like cocaine, morphine and alcohol and in those subjected to stressors, including CMS and pre-natal capsaicin exposure. In the mouse model, the *CB2* gene transcripts were present in whole brain preparations following CMS and CB2-R protein was enhanced by CMS and prenatal capsaicin exposure. Chronic treatment with CB2-R agonist JWH015 enhanced alcohol consumption in stressed but not in control mice. In animals that developed alcohol preference, *CB2* gene expression was down regulated in the midbrain and striatum suggesting the involvement of CB2-Rs in the effects drugs of abuse.

*CB2* gene structure has been poorly defined and characterized and less well studied for CNS function unlike the *CB1* gene [27]. However, many features of the *CB2* gene structure, regulation and variation are beginning to emerge with identification of neuronal CB2-Rs in CNS [27], [28]. The human *CB2* gene consist of a single translated exon [29], and single untranslated exon and similar *CB2* gene structure is in mice but encodes two transcripts using different first exons. Most regions of the *CB2* gene are highly conserved, but the human has glutamine and mice and rats have arginine at position 63 [29]. In humans a number of polymorphisms in the *CB2* gene including Q63R [28], [29] and H316Y have been linked to osteoporosis and autoimmune disorders. We tested the hypothesis that genetic variants of the *CB2* gene might have significant effects and association with depression and alcoholism. This hypothesis is supported by the identification of a missense polymorphism at *CB2* cDNA position 188–189, which results in a dinucleotide conversion of AA to GG and predicts a non conservative amino acid substitution of glutamine by arginine at position 63 (Q63R). This tandem polymorphism is important as it has the potential to change function in the mature expressed cannabinoid CB2-R as demonstrated in the immune system by an *in vitro* assay [29]. The association of *CB2* gene variation was probed in Japanese subjects to examine the non-synonymous polymorphism, Q63R, in the *CB2* gene for association with depression or alcoholism. There was significant difference in allelic frequency between cases and controls at Q63R polymorphism in the *CB2* gene in depression in this study and alcoholism [19]. As many genetic variants play various roles in depression and/or substance abuse, variation in *CB2* gene, (Q63R) polymorphism may be a previously unknown risk factor in depression and/or alcoholism at least in the Japanese population. If this can be generalized to other ethnicities, then the results support the possibility of targeting the cannabinoid system using CB2-R ligands in depression and drug abuse and perhaps in their co-morbidity. It is therefore tempting to speculate that the reported effects of alcohol may be associated with changes in the cannabinoid system, with CB2-Rs playing a regulatory role.

We then hypothesized that if CB2-Rs are present in the brain, then antisense oligonucleotides complementary to *CB2* mRNA transcript will block translation of or stimulate degradation of *CB2* mRNA. It is therefore of importance to determine whether inhibition of *CB2* gene expression in the brain will alter behavior as observed with the exogenous administration of CB2-R ligands. Direct intracerebroventricular *CB2* oligonucleotide microinjection into the mouse brain reduced mouse aversion, further indicating the functional presence of CB2-Rs in the brain that influence behavior. This behavioral evidence for the functional presence of CB2-Rs in brain was further investigated by the exogenous administration of CB2-R agonists and antagonist. The behavioral effects of acute treatment with JWH015, a CB2-R agonist and SR144528, a CB2-R antagonist, in mouse spontaneous locomotor activities, and in the two- compartment black and white box lends further support that CB2-Rs in the brain modifies behavior. Similar observations have been reported for the effects of a CB2-R agonist, GW405833 [25]. Curiously, the observation that CB2-R agonists induces sedation and catalepsy only at higher doses has been interpreted by this group and others in rodent models of pain, to have a potential to treat pain without eliciting the centrally-mediated side effects without the psychoactivity associated with CB1-R [13], [25]. With the recent definitive demonstration of neuronal CB2-Rs in the brain, one possible explanation may be that CB2 and CB1 cannabinoid receptors work independently and/or cooperatively in different neuronal populations to regulate a number of physiological activities influenced by cannabinoids. These effects of CB2-R ligands in *in vivo* behavioral tests are provided as functional evidence of CB2-R in the brain that plays a role in motor function and emotionality tests. The antagonism of the behavioral effects of CB2-R agonist, JWH015 by SR144528 or AM630 was not determined in this study. However, other studies have demonstrated the selectivity of JWH015 on mediating its effects via CB2-Rs [30], [31] and the effect of JWH015 was completely blocked by the CB2 specific antagonist, SR144528 [32].

Abundant CB2-R immunoreactivity in neuronal and glial processes was detected but at a much lower level than CB1 receptors as reported [23]. This is supported by reports of the presence of CB2-Rs in brain stem, cortex, cerebellum, dorsal root ganglion and spinal cord [21]–[23]. There is still some controversy on the specificity of CB2-R staining because most of the antibodies are capable of producing non-specific staining. Therefore, very rigorous controls have been utilized in our study including *1*) the pre-adsorption and co-incubation of the CB2-R antibody with the immunizing peptide resulting in blocking CB2 staining in the rat cerebellum, *2*) *in situ* hybridization data show that *CB2* gene is expressed in the cerebellum of wild type and not in the CB2 knockout mice, with *CB2* gene being absent in the sense control in the wild type mice. The absence of CB2 mRNA in CB2-R deficient mice and presence in wild type controls has also been demonstrated by others [21]. Moreover, in previous control experiments we had demonstrated that using two types of CB2 antibodies, similar staining patterns in both the rat spleen and cerebellum [24] were reported. Western blot analyses revealed specific bands that were identified using CB2 antibodies and were absent when the CB2 antibodies were pre-adsorbed with the immunizing peptide [24]. Furthermore, the expression levels of the CB1 gene using RT-PCR analysis was 100 times that of the CB2 gene expression levels with reference to the brain stem. We have also confirmed that the spleen has the most abundant *CB2* gene transcripts when compared to other regions [24].

As definitive electron microscopic evidence is needed to precisely determine the subcellular localization of CB2-Rs, our transmission electron micrograph data using immunoelectron microscopy approach shows a high-resolution definition of hippocampal CB2-R localization at the ultrastructural level. Electron micrographs from hippocampal areas show dendrites with immunostaining for CB2-Rs with diffuse black deposits and mitochondria clearly visible. In some areas axon terminals were not immunoreactive for CB2-Rs and small rounded synaptic vesicles were seen. An axon terminal making contact with a dendrite but without immunostaining for CB2-Rs was apparent. The pattern of staining in most hippocampal areas appears to be mainly post-synaptic localization of CB2-Rs. For example at the area of the synaptic contacts seen the synapse appear to be excitatory and possibly glutamatergic. We cannot exclude that some of the CB2-Rs may be presynaptic, just like CB1-Rs are not exclusively presynaptic in the brain [33]. CB1-Rs are known to be mainly presynaptic in the CNS where cannabinoids act at presynaptic CB1-Rs and endocannabinoids have emerged as one of the classes of retrograde messengers involved in the regulation of synaptic transmission. The functional implication of pre- and/or post-synaptic localization of CB2-Rs awaits further electrophysiological investigation and image analysis of this interesting component of the EPCS. The current understanding of CNS CB2-Rs was the subject of our review [18] and future studies will continue to characterize the specificity of CB2-R mediated behavioral effects and their physiological roles. Thus, our data demonstrate the functional expression of CB2-Rs in brain that may provide novel targets for the effects of cannabinoids in depression and substance abuse disorders beyond neuro-immunocannabinoid activity.

## Materials and Methods

### Human subjects

166 patients with Major depression (excluded bipolar disorders) diagnosed as depressed by DSM-IIIR criteria without other psychiatric diagnoses, recruited under informed consent. 487 age- and gender-matched controls were research volunteers. They were recruited from north-mid main island area in Japan and provided written informed consent. The genetics study using the DNA of subjects, who provided written consent, was approved by ethics committee of University of Tsukuba.

### Animal subjects

Three strains (DBA/2, C57BL/6 and BALBc), male and female mice and Sprague Dawley rats were used. CB2 knockout mice (CB2^−/−^) and their wild type littermates used in this study CB2^−/−^ was developed by Buckley *et al*, 2000, [34] and obtained from the National Institutes of Health through Dr. Kunos of NIAAA-NIH, USA. Animals were housed according to National Institutes of Health and institutional guidelines for laboratory animals. All procedures were approved by the local Animal Care and Use Committees in all the institutions involved with the project.

### Drugs

JWH015 (a putative CB2 agonist) and AM60, a CB2-R antagonist were obtained from Sigma-Aldrich (MO, USA) and Cayman Chemicals (MI, USA) while SR144528 (a CB2 antagonist) was donated by Sanofi, (France). Primary CB2 antibodies and their blocking peptide were obtained from Santa Cruz (Ca, USA). For the *in vivo* experiments, JWH015, AM630 and SR144528 were made up in ethanol: emulphur: water in a ratio of 1∶1∶18.

### Behavioral Analyses

BALB/c male and female mice were housed 12 hrs in light and 12 hrs in dark. Experimental mice (N = 10 per group) were exposed to CMS everyday for four weeks to achieve anhedonia (CMS test). These experimental animals were subjected to the weekly CMS regime consisting of three 10 hr periods of 45° cage tilt; 3 periods of overnight stroboscopic illumination, two 10 hr periods of empty water bottle; two periods of overnight food or water deprivation; two 10 hr periods of damp bedding. The CMS treated and non-stressed groups consisted of 30 mice each and were split into three subgroups, respectively. All non-stressed groups were given food and water at all times, as well as comfortable cage surroundings, while the experimental group was housed in a different room. In the first set of studies animals in both the stress and control groups of 10 animals per group were treated daily with the CB2 agonist JWH015 (20 mg/kg) and the control groups with the vehicle for 4 weeks. In the second round of CMS study animals in both the stress and control groups of 10 animals per group were treated daily with the CB2 antagonist AM630 ( 1 and 3 mg/kg) and the control groups with the vehicle for 4 weeks. Once every week sucrose consumption was measured as a test of anhedonia. At the end of the stress regime, locomotor activities stereotype behavior was measured in activity monitors in all groups.

The acute effects of JWH015, a CB2-R agonist and SR144528 a CB2-R antagonist on mouse locomotor activity and stereotypy using activity monitors and in the two- compartment black and white box were assessed. The pretreatment times were 10 min- for the agonist and 30 min for the antagonist. Animals were placed in activity monitors or in the two- compartment black and white box. Spontaneous locomotor activities and stereotype behavior in the activity monitors and time spent and locomotor activities in the box were obtained from the automated system. The doses of the agonist and antagonist were 1–20 mg/kg except as indicated in specific experiments as described for the CMS experiments. The performance of mice in the plus-maze test of anxiety following intracerebroventricular (ICV) administration of CB2 antisense oligonucleotide (oligos) (20 µg in 5 µl) was assessed before and after 3 days of twice daily microinjection and compared to mice injected with sense and mismatched oligos.

### Western Blotting

Equal amount of protein 20 µg obtained from the brains of stressed and control mice were loaded and separated by 10% SDS-PAGE and then transferred onto nitrocellulose membrane. The nitrocellulose was washed and blocked in PBS containing 2% non-fat milk and incubated with the CB2 antibody overnight. The membranes were washed and incubated with a conjugated goat anti-rabbit secondary antibody and processed for immunoreactivities with and without pre-incubation of the primary antibodies with CB2 peptide.

### *CB2* gene expression and regulation by drug and alcohol treatment

CB2-R gene expression was determined in whole mouse brains subjected to stressors and those treated chronically with heroin (10 mg/kg) and cocaine (40 mg/kg) and then precisely in brain regions of naïve mice and those exposed acutely or chronically to escalating doses of alcohol. *CB2* gene expression was then determined in animals that developed or did not develop alcohol preference. Mice were sacrificed and whole brains were taken or dissected out for extraction of RNA. Control group of mice (n = 6) did not have access to ethanol but only to water in that experiment, and RNA was also extracted in a same way for comparison to the mice that developed ethanol preference. Where indicated brains were dissected into striatum, hippocampus and midbrain. RNA was extracted using RNeasy kit (QIAGEN, K.K., Tokyo, Japan) and cDNA was synthesized by Revertra Ace (TOYOBO, Japan) and oligo dT primer. The expression of *CB2* gene was compared by TaqMan real-time PCR with an ABI PRISM 7900 HT Sequence Detection System (Applied Biosystems, Foster City, CA, USA), using the TaqMan gene expression assay for *CB2* (Mm0043826_m1).

### Association study between the Q63R polymorphism and depression

*CB2* gene has two non-synonymous polymorphisms, Q63R and H316Y according to public database NCBI (http://www.ncbi.nlm.nih.gov/). However, analysis of secondary structure of *CB2* gene with Chou-Fasman, Robson and hydrophilic/ hydrophobic structure extimation methods using computer program GENETYX (Genetyx corporation, Tokyo, Japan) revealed a potential structural change in *CB2* gene only by the Q63R but not by the H316Y polymorphism of the gene. Therefore, we focused on the Q63R polymorphism and the genotype was determined by restricted fragment length polymorphism (RFLP) method as described in our previous report [19].

### Real Time-PCR

Total RNA was extracted from brain tissues using RNAzol B (Tel-Test, Friendswood, TX). Single strand cDNA was synthesized from total RNA using SuperScript™ first-strand synthesis system for RT-PCR (GIBCO/BRL, Rockville, MD). For quantitative real time PCR assays, the exon-specific primers and fluorescent Fam-labeled probes across different exon regions were designed using Primer Express program (Applied Biosystems, Foster City, CA). Beta-actin Fam-labeled probe was used for normalization. Two-step PCR program was used as the default of ABI 7900 HT PCR instrument (Applied Biosystems, Foster City, CA). In the assay, we used spleen as positive control because of its high expression of *CB2* gene and no cDNA in the TaqMan PCR reaction as negative control. The control brain samples were set at 1.0 with glyceraldehydes-3-phosphate dehydrogenase (GAPDH) pre-developed TaqMan assay reagent as endogenous control (FAM™ Dye/MGB probe). Calculation of real time PCR was carried out according to User Bulletin #2 for ABI Prism 7700 Sequence Detection System.

### Immunohistochemistry and electron microscopy

Mice and male Sprague Dawley (180–200 g) rats were anesthetized with choral hydrate (300 mg/kg) pentobarbital, perfused transcardially with saline and then with 100 ml of 4% paraformaldehyde in phosphate buffer (PB; 0.1 M, pH 7.4) for mice and 500 ml of the same fixative solution for rats. Brains were dissected, postfixed in the same fixative solution for 2 hours at room temperature, equilibrated with 30% sucrose in PB at 4°C, frozen and cut into saggital 20–40 µm sections using a cryostat. Sections were processed for immunohistochemistry as follows. Floating sections were incubated with 1% hydrogen peroxide in phosphate-buffered saline (PBS) for 10 min at room temperature to inhibit endogenous peroxidase, washed three times with PBS, incubated for 1 h in 3% normal goat serum (NGS) in Tris-buffered saline (TBS), pH 7.4 at 22°C, incubated in primary CB2 antibody obtained from (Santa Cruz, Ca, USA), diluted 1: 300 in TBS containing 3% NGS for 24 h at 4°C, rinsed, incubated for 1 h at 22°C in 1∶200 dilution of biotinylated goat anti-rabbit secondary antibody (Vector, Burlingame, CA, USA) for 1 h, rinsed, incubated with avidin-biotin peroxidase complex (ABC) reagent for 1 h (Vector), rinsed, and then incubated in a solution containing 22 µg/ml diaminobenzidine (DAB) (Electron Microscopy Sciences, Fort Washington, PA) and 0.003% hydrogen peroxide (H_2_O_2_) for color deposition. Sections were mounted on coated slides, dehydrated, cover slipped, viewed and photographed using Zeiss and Leitz microscope and a Nikon digital camera, immunoreactive elements plotted onto the atlas depictions [35], and images edited using photoshop (vCS; Adobe systems). As additional control, iCB2 of brain sections from CB2-R deficient mice and wild type controls were also analyzed. For electron microscopy, rats were perfused with 500 ml of 4% paraformaldehyde, 0.1% purified glutaraldehyde fixative in PB, brains were removed, postfixed in the same fixative solution for two hours and saggital sections (50 µm) were obtained using a vibrotome. Then, sections were processed for inmmunohistochemistry following the same immunoperoxidase protocol. After that, sections were fixed with 1% osmium tetroxide in 0.1 M PB for 1 h, dehydrated through a series of graded alcohols (including 60 min in 70% alcohol containing 1% uranyl acetate), and then with propylene oxide. Afterwards, they were flat-embedded in Durcupan ACM epoxy resin (Electron Microscopy Sciences, Fort Washington, PA). Embedded sections were polymerized at 60°C for 2 days. Ultrathin sections of 70 nm were cut from the outer surface of the tissue with an ultramicrotome (Leica, Microsystems, Wetzlar, Germany) using a diamond knife (Diatome, fort Washington, PA). The sections were collected onto 300 mesh cooper grids and counterstained with Reynolds lead citrate [36]. Sections were examined and photographed using a Zeiss 109 transmission electron microscope and 35 mm Kodak technical Pan professional 2415 films.

### *In situ* hybridization and probes

Biotin labeled RNA probes were used for *in situ* hybridization. The full length of human *CB2* gene was subcloned from pcDNA3.1/CB2 (UMR cDNA resource center, Rolla, MO) into pBluescript II at the restriction sites of EcoR I and Xho I. The pcBluescript II/CB2 was linearized with Xho I (Anti-sense probe) or Eco RI (sense probe). The CB2 riboprobes were synthesized by incubating for 60 min at 37°C. 1 µg linearized plasmid in 2 µl 10X transcription buffer, 1 µl RNase inhibitor, 2 µl Biotin RNA labeling Mix containing 1 mM ATP/GTP/CTP, 650 µM UTP, 350 µM biotin–UTP (Roche Applied Science, Germany), 40 U T7 (anti-sense probe), or T3 RNA polymerase (sense probe) in a final volume of 20 µl. The reaction mixture was subsequently incubated for 15 min at 37°C with 1 U RNase-free DNase I. The riboprobes were precipitated using LiCl and ethanol. The CB2 probes were diluted in 100 µl TE. Coronal cerebellum sections (20 µm) of wild type and CB2 knock-out mouse were cut in a cryostat microtome. All solutions were prepared in deionized H_2_O treated with 0.1% (V/V) diethylpyrocarbonate and autoclaved. Sections were incubated with 1% hydrogen peroxide in phosphate buffered saline (PBS) for 10 minutes at room temperature to inhibit endogenous peroxidase, washed three times with PBS. Sections were fixed by immersion in 4% paraformaldehyde in PBS, pH 7.4, and then briefly rinsed twice with PBS. After treatment with Proteinase K, sections were refixed in 4% paraformaldehyde. The sections then were acetylated by immersion in 0.1 M triethanolamine containing 0.25% acetic anhydride, permeabilized by 1% Triton X-100, and rinsed twice with PBS. Prehybridization was carried out at 4°C overnight with prehybridization solution (50% formamide, 4×SSC, 0.5× Denhardt's solution, 100 mM DTT, 250 µg/ml yeast tRNA, and 250 µg/ml salmon sperm DNA). For hybridization, the sections were incubated in a prehybridization solution containing 1 µg/ml of cRNA probe, incubated at room temperature overnight on a shaker. Sections were immersed sequentially in 0.2× SSC twice and buffer 1(0.1 M tris pH 7.5, 0.15 M NaCl) twice. The sections were incubated with ABC reagent for 1 hour (Vector), rinsed, and then incubated with diaminobenzidine for color deposition.

### Statistical analysis

Data for motor function tests and emotionality tests were analyzed by analysis of variance for multiple comparisons followed by Turkey's test where appropriate. The accepted level of significance is p<0.05. For *CB2* gene expression analysis, unpaired *t* test (GraphPad software) was used and p<0.05 is the accepted level of significant difference. Deviations of the observed allele and genotype distributions from Hardy-Weinberg equilibrium (HWE), were calculated by HWE computer program, and differences in allele frequencies between case-control groups were tested for significance using Fisher's exact tests on 2×2 contingency tables.
